# Validity of V̇O_2max_ estimates from the forerunner 245 smartwatch in highly vs. moderately trained endurance athletes

**DOI:** 10.1007/s00421-025-05923-x

**Published:** 2025-08-06

**Authors:** Florian A. Engel, Lukas Masur, Billy Sperlich, Peter Düking

**Affiliations:** 1 Integrative and Experimental Exercise Science and Training, Institute of Sport Science, Julius-Maximilians-Universität Würzburg, Judenbühlweg 11, 97082 Würzburg, Germany; 2 Department of Sports Science and Movement Pedagogy, Technische Universität Braunschweig, Braunschweig, Germany

**Keywords:** V̇O_2max_ assessment, Endurance athletes, Smartwatch, Smart technology, Physiological diagnostics, Running performance

## Abstract

**Purpose:**

Assessing the validity of maximum oxygen uptake (V̇O₂_max_) estimates provided by a commercially available smartwatch (Garmin Forerunner 245, Garmin Ltd., Olathe, USA) compared to laboratory-based respiratory gas analysis in moderately-to-highly trained athletes.

**Methods:**

Thirty-five endurance athletes (Tier 2–3 athletes, 24 males, 11 females; age: 25.1 ± 3.5 years; V̇O₂_max_: 60.1 ± 8.2 ml·min⁻^1^·kg⁻^1^) completed a treadmill ramp test with respiratory gas analysis to determine criterion V̇O₂_max_. Additionally, each athlete performed two submaximal 15-min outdoor runs at > 70% of maximum heart rate, during which the smartwatch estimated V̇O₂_max_. Athletes were stratified into moderately trained (V̇O₂_max_ ≤ 59.8 ml·min⁻^1^·kg⁻^1^) and highly trained (V̇O₂_max_ > 59.8 ml·min⁻^1^·kg⁻^1^) subgroups.

**Results:**

Across all athletes, the smartwatch underestimated V̇O₂_max_ [mean differences: − 4.73 ml·min⁻^1^·kg⁻^1^ (run 1), -4.05 ml·min⁻^1^·kg⁻^1^ (run 2)]. Intraclass correlation coefficients (ICC) indicated moderate agreement between smartwatch and laboratory values (run 1: ICC = 0.71 [95% CI: 0.03–0.90]; run 2: ICC = 0.75 [95% CI: 0.17–0.91]), with mean absolute percentage errors (MAPE) of 7.9% and 7.2%. Subgroup analyses revealed better accuracy of smartwatch estimated V̇O₂_max_ in moderately trained group (MAPE: 4.1–2.8%; ICC: 0.63–0.66 [95% CI: 0.09–0.87]), whereas in highly trained athletes, the smartwatch underestimated V̇O₂_max_ by 6.3 ml·min⁻^1^·kg⁻^1^ (MAPE: 10.4–9.4%; ICC: 0.34–0.41 [95% CI: − 0.11–0.75]).

**Conclusion:**

Smartwatch-derived V̇O₂_max_ estimates are valid in moderately trained athletes but less valid in highly trained individuals. While smartwatches are useful for general monitoring, caution is warranted in their interpretation, particularly in highly trained individuals. Laboratory-based gas analysis remains the preferred method when precision is required.

## Introduction

Maximal oxygen uptake (V̇O₂_max_) constitutes a central indicator of aerobic capacity and cardiorespiratory fitness (Bassett and Howley 2000). It refers to the highest rate at which the cardiorespiratory system can deliver oxygen to the working muscles during intense exercise, and the working muscles' capacity to utilize this oxygen for energy production via oxidative metabolism (Bassett and Howley 2000). In endurance disciplines such as long-distance running, elevated V̇O₂_max_ values are strongly associated with competitive success (Joyner et al. 2020; Jones et al. 2021; Lucia et al. 2008). Beyond its relevance to athletic performance, V̇O₂_max_ serves as a robust marker of cardiovascular health and exhibits an inverse relationship with morbidity and all-cause mortality, underscoring its significance for health and longevity (Mandsager et al. 2018). Accordingly, V̇O₂_max_ assessment enables the quantification of physiological fitness, facilitates retrospective evaluation of training efficacy, and informs prospective training prescription for both performance enhancement and health promotion (Bassett and Howley 2000; Levine 2008; Kodama et al. 2009Mandsager et al. 2018).

Currently, the gold standard for assessing V̇O₂_max_ in athletes involves ramp-incremental exercise protocols performed on a treadmill until volitional exhaustion, combined with breath-by-breath respiratory gas analysis (Tran 2018; Poole and Jones 2017b). Although this method yields high physiological validity, it is time-intensive, costly, and requires access to specialized equipment and trained personnel. In addition, the requirement for maximal exertion can interfere with athletes’ training and recovery schedules, particularly during periods of high load or competition. Consequently, practitioners frequently regard ramp testing as impractical for routine application in practical settings (Schimpchen et al. 2023).

As an alternative to laboratory-based maximal cardiorespiratory fitness tests, the estimation of V̇O₂_max_ using commercially available smartwatches has gained increasing attention in recent years (Sperlich and Holmberg 2017; Sperlich et al. 2019; Düking et al. 2022). These wearable devices estimate V̇O₂_max_ during outdoor running sessions at submaximal intensities, provided that heart rate and Global Positioning System (GPS) data are continuously recorded (Garmin Ltd. 2022). The estimation is presumably based on variables, such as heart rate, running speed, and distance, which are processed through proprietary algorithms. However, the exact input parameters and computational models employed for these estimations remain undisclosed, limiting transparency and validation opportunities for both athletes and researchers, as it is unclear how factors such as participants training level (next to others, such as intensity of session, pace, etc.) affect smartwatch-derived V̇O₂_max_ estimations.

Several studies have examined the validity of smartwatch-derived V̇O₂_max_ estimates. For instance, Molina-García et al. (Molina-Garcia et al. 2022) reported a mean bias of – 0.09 ml·kg⁻^1^·min⁻^1^, with limits of agreement ranging from –9.92 to 9.74 ml·kg⁻^1^·min⁻^1^. However, the accuracy of V̇O₂_max_ estimations obtained from wearable devices, such as smartwatches and smartphones, appears to vary across individuals with different levels of aerobic capacity (Düking et al. 2022, 2024; Helgerud et al. 2022). Notably, several investigations have indicated a systematic underestimation of V̇O₂_max_ in sedentary individuals with elevated aerobic capacity (Helgerud et al. 2022; Carrier et al. 2023) and athletes with higher V̇O₂_max_ (Düking et al. 2024, 2022). As exact algorithms and methodologies are not disclosed by the manufacturer, it is unclear why different results for validity are derived when assessing V̇O₂_max_ with smartwatches in different populations. Nonetheless, this suggests that device validity may decrease with increasing levels of cardiorespiratory fitness. This discrepancy likely reflects limitations inherent in the algorithms and sensor technologies employed, which may lack sufficient sensitivity to accurately capture the physiological characteristics typical of highly trained athletes. Despite the increasing use of smartwatches for estimating V̇O₂_max_, no studies have specifically examined their validity in individuals with V̇O₂_max_ values exceeding 60 ml·min⁻^1^·kg⁻^1^ and only two studies to date have examined athletes with V̇O₂_max_ values above 55 ml·min⁻^1^·kg⁻^1^ (Düking et al. 2024; Carrier et al. 2023). To the best of our knowledge, no study has conducted a dedicated subgroup analysis comparing athletes above a V̇O₂_max_ of 60 ml·min⁻^1^·kg⁻^1^.

Accordingly, the present study pursues the following aims: (i) to validate the V̇O₂_max_ estimates provided by the Forerunner 245 smartwatch (Garmin, Olathe, USA) against laboratory-based gold-standard assessments, and (ii) to determine whether the smartwatch systematically underestimates V̇O₂_max_ in highly trained endurance athletes (V̇O₂_max_ > 60 ml·min⁻^1^·kg⁻^1^) to a greater extent than in moderately trained endurance athletes. Based on previous investigations into smartwatch- and smartphone-derived V̇O₂_max_ estimations in heterogeneous populations (Düking et al. 2022, 2024; Carrier et al. 2023; Helgerud et al. 2022), we hypothesized that (i) the average estimation error would range between 5 and 10%, and (ii) the estimation error would be significantly greater in highly trained athletes compared to their moderately trained counterparts. The Garmin Forerunner 245 (Garmin Ltd., Olathe, USA) was selected for this study based on its integrated capability to estimate maximal oxygen uptake (V̇O₂_max_) during and/or after outdoor running sessions. Garmin is among the leading global manufacturers in the smartwatch market (Statista Market Insights 2024), which underscores the practical relevance and potential generalizability of the findings to both scientific and applied contexts. The Forerunner 245 represents a mid-range model in terms of price and functionality, making it particularly relevant for a broad spectrum of recreational and competitive users. Prior research has indicated that this device tends to underestimate V̇O₂_max_, especially in individuals with higher aerobic fitness compared to moderately trained counterparts (Düking et al. 2024). Building on this, the present study aimed to further examine the accuracy of V̇O₂_max_ estimations in a sample of athletes with a higher level of aerobic training than those previously investigated.

## Methods

### Participants

A total of 35 moderately-to-highly trained endurance sports athletes (24 males and 11 females) participated in the study (mean ± SD; age: 25.1 ± 3.5 years; body height: 177.5 ± 8. cm, body mass 73.8 ± 10.7 kg). The participants’ sport modalities included distance running and triathlon and athletes were classified as Tier 2–3 athletes according to the framework proposed by McKay et al. 2022, and further categorized into performance levels based on established criteria by Decroix et al. (2016) and Pauw et al. (2013): performance level 2 (*n* = 9), performance level 3 (*n* = 10), performance level 4 (*n* = 9), and performance level 5 (*n* = 7) (Pauw et al. 2013; Decroix et al. 2016). The criteria for inclusion, assessed by questionnaire, were: 1) healthy, no chronic or acute medical condition (e.g., musculoskeletal or cardiovascular systems); 2) active athletes, classified as Tier 2–5 athletes (McKay et al. 2022). Athletes were recruited from a local triathlon club, a university sports club, and university-based sport science courses. All participants reported engaging in 3–15 training sessions per week, corresponding to a weekly training volume ranging from 5 to 20 h. They regularly competed at regional or national levels or were recreationally active with structured training regimens. None of the participants reported any acute or chronic injuries or medical conditions at the time of data collection. Prior to participation, all individuals were fully informed about the study procedures and provided written informed consent. Participation was voluntary, and all individuals were free to withdraw from the study at any point without consequence. The local ethics committee approved the study design without any restrictions and assigned the following reference number to the ethics application: EV2025/2–2504.

Based on similar studies with trained athletes, revealing poor-to-moderate ICCs between smartwatch-derived VO_2max_ estimations and respiratory gas analysis (Düking et al. 2024), we performed an a priori sample size calculation using Arifin’s web-based sample size calculator (Arifin 2018) with parameters set as follows: ICC, ρ0 = 0.70 (Nunnally 1978); ρ1 = 0.50 (based on previous study (Düking et al. 2024)), α = 0.05, 1–β = 0.80, k = 2, dropout = 10%. A final sample size of 79 participants was initially calculated. However, due to the limited availability of moderately and especially highly trained endurance athletes, as well as the challenges in recruiting and motivating them to participate in time-consuming research studies, the final sample included 35 athletes (Table 1).

**Table 1:** Means ± SD [range] of anthropometric parameters, training volume, and training history of participating athletes (*n* = 35)

Parameter	All athletes	Moderately trained athletes (V̇O_2max_ < 60 ml∙min^−1^∙kg^−1^ (n = 18))	Highly trained athletes V̇O_2max_ > 60 ml∙min^−1^∙kg^−1^ (n = 17)
Age [years]	25.1 ± 3.5	23.9 ± 1.5 [21–26]	26.3 ± 4.4 [19–35]
Body height [cm]	177.5 ± 8.7	175.1 ± 8.7 [156–192]	180.1 ± 8.1 [161–191]
Body mass [kg]	73.8 ± 10.7	73.7 ± 11.7 [55–93]	73.9 ± 9.6 [52–89]
Training sessions per week [n]	6.2 ± 4.2	4.2 ± 2.0 [2–10]	9.2 ± 4.9 [3–15]
Hours of training per week [h]	9.7 ± 4.6	7.6 ± 2.6 [4–14]	13.5 ± 5.1 [6–20]
Accumulated years of training [years]	10.4 ± 5.6	12.9 ± 5.5 [3–20]	7.8 ± 4.4 [3–17]
V̇O₂_max_ [ml∙min^−1^∙kg^−1^]	60.1 ± 8.2	53.6 ± 5.1 [43–60]	67.0 ± 4.3 [60–75]

### Experimental overview

The primary objective of this study was to evaluate the accuracy of V̇O₂_max_ estimates generated by the Forerunner 245 smartwatch (Garmin, Olathe, USA) based on two submaximal 15-min outdoor runs, in comparison to the criterion measure of V̇O₂_max_ obtained via respiratory gas analysis during a treadmill ramp test. This comparison was conducted separately for highly trained and moderately trained endurance athletes. Data collection for the study took place between June and July 2024. During this period, the athletes were either in their competitive season or shortly before its onset. To ensure standardized testing conditions, all athletes were instructed to arrive in a rested state, with a recovery day scheduled prior to each testing session.

During the initial laboratory visit, baseline data were collected, including anthropometric characteristics, training history and volume, and screening for inclusion and exclusion criteria. V̇O₂_max_ and maximal heart rate (HRₘₐₓ) were assessed using a ramp-incremental treadmill protocol to volitional exhaustion, with simultaneous respiratory gas analysis via a metabolic cart equipped with a mixing chamber.

**Fig. 1:**
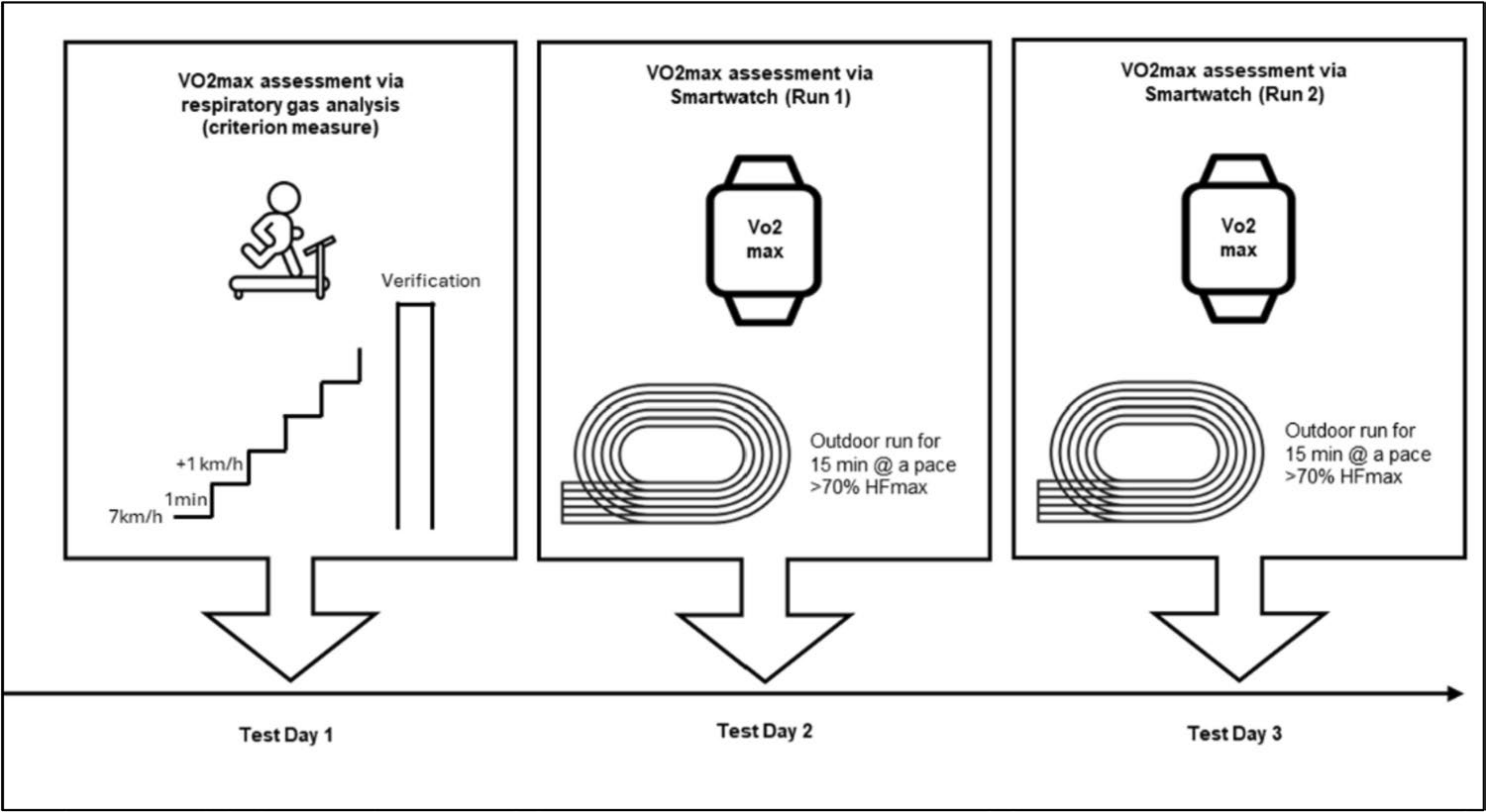
Illustration of experimental procedures

Subsequently, participants completed two 15-min submaximal running sessions on non-consecutive days, with rest intervals of 48–72 h. These runs were performed on a 400-m outdoor track at an exercise intensity exceeding 70% of individually determined HRₘₐₓ. During each run, participants wore the Forerunner 245 smartwatch with the integrated GPS and optical heart rate sensors activated to capture real-time physiological and performance data. An overview of the experimental protocol is presented in Fig. 1.

### Assessment of maximum oxygen uptake using respiratory gas analysis

All participants completed a ramp-incremental treadmill test followed by a verification phase to determine maximal oxygen uptake (V̇O₂_max_). Testing was performed on a motorized treadmill (Mercury, h/p/cosmos sports and medical GmbH, Nussdorf-Traunstein, Germany). The incremental treadmill protocol commenced at a running speed of 7 km·h⁻^1^ with a 1% incline. Speed was increased by 1 km·h⁻^1^ every minute until volitional exhaustion. In cases where participants completed the maximal running velocity of 22 km·h⁻^1^, the incline was subsequently increased by 1% per minute until exhaustion. This extension of the protocol was necessary in three participants.

Exhaustion and V̇O_2max_ were considered valid when participants met at least three of the following four criteria: (1) a plateau in oxygen uptake, defined as an increase of < 1.0 mL·min⁻^1^·kg⁻^1^ despite an increase in treadmill speed; (2) a respiratory exchange ratio (RER) > 1.10; (3) a rating of perceived exertion (RPE) > 18 on the Borg scale; and (4) a peak blood lactate concentration (LAₚₑₐₖ) > 6 mmol·L⁻^1^. Given ongoing debate regarding the sufficiency of these criteria to confirm V̇O₂_max_, a verification trial was conducted 5 min following the completion of the ramp test, in line with the established recommendations (Poole and Jones 2017a; Schaun 2017). This trial was performed at a speed 1 km·h⁻^1^ above the final stage of the ramp protocol, with participants receiving verbal encouragement to continue for as long as possible. V̇O₂_max_ values from the incremental step test and the verification trial were compared, and the higher value was used for further analysis (Schaun 2017).

Throughout both the ramp and verification trials, respiratory gas exchange was continuously measured using a dynamic micro-mixing chamber system (Q-NRG MAX, Cosmed, Rome, Italy), which served as the criterion method for determining V̇O₂_max_. The metabolic measurements were conducted using the Q-NRG MAX metabolic cart, which uses a micro-dynamic mixing chamber system. This system samples respiratory gases during each exhalation, proportionally aligned with the athlete’s expiratory flow. The sampled gas is directed into a micro-dynamic mixing chamber, where the concentrations of oxygen and carbon dioxide are analyzed. Because the gas sample entering the chamber mirrors the athlete’s expiratory pattern on a reduced scale, the system can compute both the average volume and mean concentrations of the exhaled gases. By combining these measurements with minute ventilation, the device calculates oxygen uptake (V̇O₂) and carbon dioxide output (V̇CO₂). The Q-NRG MAX outputs averaged gas concentration values every 30 s (COSMED 2024; Priem et al. 2023). The highest 30-s averaged value for oxygen uptake (VO₂) recorded during the incremental treadmill test was defined as VO₂_max_. The gas analyzer was calibrated prior to each test using a precision 3-L syringe and a calibration gas mixture (16% O₂, 5% CO₂ in N₂; Cosmed, Rome, Italy), selected to match the expected range of fractional gas concentrations. This system has demonstrated high accuracy and reliability for the measurement of ventilation, oxygen consumption, and carbon dioxide production across a wide range of metabolic rates (Falcioni et al. 2025).

Heart rate was continuously monitored throughout both the ramp protocol and the subsequent verification trial using a chest strap (Polar H10, Polar Electro OY, Kempele, Finland) compatible with the cosmed Q-NRG MAX. The continuously measured heart rate was transmitted to the metabolic cart via Bluetooth technology, allowing real-time synchronization of heart rate measurements with the metabolic cart system. For the final analysis, 30-s mean heart rate values were extracted and used. Heart rate at the time of V̇O_2max_ was considered as maximum heart rate (HR_max_).

### Protocol for assessment of maximum oxygen uptake with the smartwatch

For the present investigation, we employed a multi-sensor, wrist-worn smartwatch (Forerunner 245, Garmin, Olathe, USA; software version 13.00). This device was selected due to its capability to provide V̇O₂_max_ estimations during outdoor running sessions. Additionally, Garmin is recognized as one of the leading global manufacturers in the smartwatch market, which enhances the relevance and generalizability of the findings for both scientific and applied contexts. (Statista Market Insights 2024). The Forerunner 245 smartwatch is equipped with an optical heart rate (HR) sensor and a Global Positioning System (GPS) receiver and can be optionally paired with an external HR chest strap for improved accuracy. To simulate a real-world application scenario, the smartwatch was configured and operated according to the manufacturer’s guidelines. Individual anthropometric characteristics, including age, sex, body height, body mass, and laboratory-determined maximal heart rate (HRₘₐₓ), were entered into the device prior to testing.

Although the manufacturer does not disclose the specific algorithms used to estimate V̇O₂_max_, it is stated that the estimation relies on submaximal outdoor running sessions lasting at least 10 min, during which heart rate must exceed 70% of HRₘₐₓ for several minutes. The device processes HR and GPS-derived velocity data from these sessions to estimate V̇O₂_max_. This approach reflects typical use conditions in athletic practice and aligns with the manufacturer's intended application of the technology (Garmin Ltd. 2022)_._ Each participant completed a 15-min continuous run on a 400-m outdoor track while wearing the Forerunner 245 smartwatch. Running pace was individually adjusted to ensure that heart rate remained consistently above 70% of the participant’s laboratory-determined HRₘₐₓ. Heart rate was continuously measured via a Garmin-compatible chest strap (Polar H10, Polar Electro Oy, Kempele, Finland) which was shown to provide valid heart rate recordings throughout a wide range of activities (Gilgen-Ammann et al. 2019), with real-time HR data displayed on the smartwatch. Investigators supervised each run and monitored HR to verify that the targeted HR was maintained throughout the session. For the final analysis, heart rate data recorded during the 15-min run were averaged over the entire duration.

To reduce external distractions and pacing influences, athletes performed the runs individually and were accompanied by an investigator. According to the manufacturer’s guidance, V̇O₂_max_ estimation accuracy may improve after multiple sessions “a couple of runs” (Garmin Ltd. 2022). Therefore, each athlete completed a second 15-min run under the same conditions approximately 48–72 h after the initial session.

### Statistical analysis

To investigate the validity of the V̇O_2max_ provided by the smartwatch, we compared values of the smartwatch against the respiratory gas analysis during the ramp protocol on the treadmill using different statistical metrics: i) mean absolute percentage error, ii) Bland–Altman analysis, iii) Intraclass Correlation Coefficients (ICC), and iv) Student’s t test for paired samples.

Data were checked for normal distribution using the Kolmogorov–Smirnoff test which revealed normal distribution for all variables (*p* ≥ 0.05).

To provide an indicator of the overall measurement error, mean absolute percent errors (MAPE) were calculated as previously performed (Lee et al. 2014). We followed previously performed statistical analysis to investigate validity to increase comparability of results between studies (Düking et al. 2024).

MAPE was calculated as average of absolute difference between the smartwatch and the respiratory gas analysis divided by the respiratory gas analysis values, multiplied by 100. The MAPE was calculated as it is a more conservative estimate of error that takes into account both over- and underestimation (Lee et al. 2014).

As previously performed (Mayorga-Vega et al. 2024), we calculated an ICC using a two-way random-effects model with absolute agreement and single measurements [also known as ICC (2.1)] (Koo and Li 2016) and was interpreted as follows: < 0.5 poor, 0.5–0.75 moderate, 0.75–0.09 good, and 0.90–1.00 excellent (Koo and Li 2016).

Bland–Altman plots were used to calculate the average difference and corresponding 95% limits of agreement.

A paired samples Student’s t test was conducted to compare the V̇O_2max_ estimates from the smartwatch with the V̇O_2max_ values obtained from respiratory gas analysis for both run 1 and run 2 in each of the two subgroups (Cohen 1988).

To assess the reliability between run 1 and run 2 for the main outcome parameters, we calculated the intraclass correlation coefficient (ICC) using a two-way mixed-effects model with absolute agreement and single measurements. ICC values were interpreted according to Koo and Li (2016).

Additionally, the V̇O_2max_ values from the two different measuring procedures were evaluated by calculating the effect size Cohen’s *d* (difference between the means/pooled SD; Cohen’s d, *d*) to estimate practical relevance, with *d* ≥ 0.2 indicating small, *d* ≥ 0.5 medium, and *d* ≥ 0.8 large effects (Cohen 1992). Cohen’s d effect sizes for unpaired samples were calculated, since the Cohen’s d effect size calculation for paired samples tends to yield very high effect sizes, which may lead to an overestimation of effect sizes (Lakens 2013). The significance level for these analyses was set a priori at *p* < 0.01.

### Subgroup analysis based on V̇O_2max_ median value

To enable subgroup analysis, the cohort of 35 athletes was stratified into two groups based on their V̇O₂_max_ values, as determined by respiratory gas analysis during the laboratory treadmill test. The distribution of V̇O₂_max_ values was analyzed, and the median value of 59.8 mL·kg⁻^1^·min⁻^1^ was used as the threshold for group allocation. Accordingly, athletes with a V̇O₂_max_ ≤ 59.8 mL·kg⁻^1^·min⁻^1^ were classified as moderately trained (*n* = 18), while those with a V̇O₂_max_ > 59.8 mL·kg⁻^1^·min⁻^1^ were classified as highly trained (*n* = 17). This median-based stratification facilitated a clear and systematic comparison between athletes with differing levels of cardiorespiratory fitness.

## Results

Table 2 depicts the results of the assessment of V̇O_2max_ with respiratory gas analysis during ramp protocol on the treadmill.

**Table 2:** Main variables (mean ± SD) obtained during and following the respiratory gas analysis during the ramp protocol on the treadmill in participating athletes (*n* = 35)

Parameter	All athletes (*n* = 35)	Moderately trained athletes (V̇O_2max_ < 60 ml∙min^−1^∙kg^−1^ (*n* = 18))	Highly trained athletes V̇O_2max_ > 60 ml∙min^−1^∙kg^−1^ (*n* = 17)
V̇O_2max_ respiratory gas analysis [ml∙min^−1^∙kg^−1^]	60.1 ± 8.2	53.6 ± 5.1	67.0 ± 4.3
RER at V̇O_2max_ [a.u.]	1.3 ± 0.2	1.2 ± 0.2	1.3 ± 0.2
Blood lactate concentration after ramp test protocol [mmol/l^−1^]	9.0 ± 2.4	9.7 ± 2.4	8.3 ± 2.2
Heart rate at V̇O_2max_ [bpm]	193.1 ± 7.6	191.2 ± 9.0	195.1 ± 5.0
Blood lactate concentration after verification trial [mmol/l^−1^]	10.5 ± 3.2	11.0 ± 2.8	8.8 ± 3.7
Rates of perceived exertion at V̇O_2max_ [a.u.]	19.0 ± 0.6	19.1 ± 0.7	18.9 ± 0.5
Running velocity at V̇O_2max_ [km·h⁻^1^]	18.8 ± 2.3	17.5 ± 2.0	20.1 ± 1.7

*RER* respiratory exchange ratio; V̇O_2max_ maximal oxygen consumption

Table 3 depicts the results of the two submaximal 15-min outdoor runs performed by the athletes while wearing the smartwatch.

**Table 3:** Duration, covered distance, mean heartrate (% maximum heart rate), and smartphone-derived V̇O_2max_ estimation in run 1 and run 2 in participating athletes (mean ± SD; *n* = 35) and intraclass correlation coefficient for main outcome parameters in run 1 and run 2

Group	Run	Duration [s]	ICC [95% CI]	Distance [m]	ICC [95% CI]	% Maximum heart rate [bpm]	ICC [95% CI]	Smartwatch V̇O_2max_ [ml∙min^−1^∙kg^−1^]	ICC [95% CI]
All athletes (n = 35)	Run 1	915.1 ± 29.5	0.923 [.829; .965]	2694 ± 775	0.636 [.098; .855]	79.0 ± 5.5	0.936 [.841; .975]	55.4 ± 6.5	0.990 [.970; .996]
	Run 2	910.8 ± 23.4		2528 ± 599		80.0 ± 5.9		56.1 ± 6.6	
Moderately trained athletes V̇O_2max_ < 60 ml∙min^−1^∙kg^−1^ (n = 18)	Run 1	917.7 ± 31.5	0.941 [.831; .979]	2629 ± 808	0.535 [− .273; .835]	76.2 ± 4.3	0.909 [-.747; .968]	50.9 ± 4.8	0.981 [.932; .994]
	Run 2	921.1 ± 39.9		2397 ± 514		77.1 ± 5.0		51.6 ± 4.9	
Highly trained athletes V̇O_2max_ > 60 ml∙min^−1^∙kg^−1^ (n = 17)	Run 1	923.2 ± 32.5	0.849 [.402; .962]	2953.8 ± 391.8	0.967 [.667; .998]	79.4 ± 6.4	0.989 [.865; .999]	60.2 ± 4.3	0.981 [.933; .994]
	Run 2	927.1 ± 22.2		3050.5 ± 553.6		79.7 ± 5.8		60.8 ± 4.6	

*ICC* intraclass correlation coefficient, *CI* confidence interval

### All athletes

**Fig. 2:**
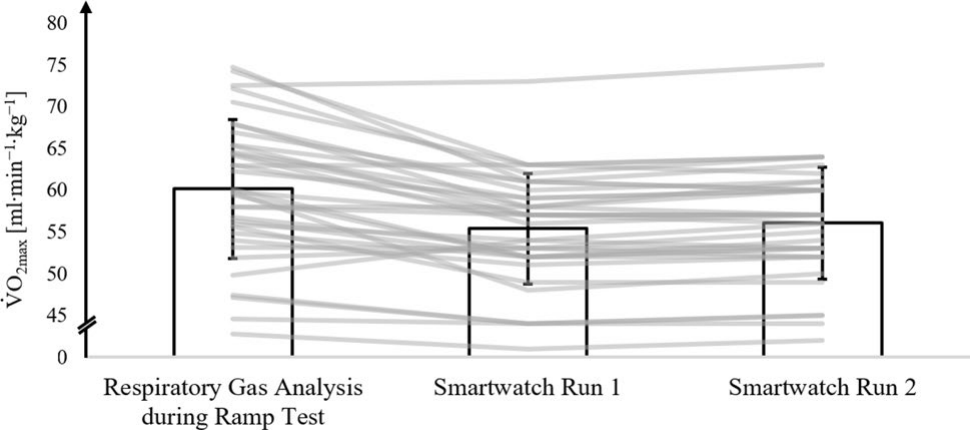
V̇O_2max_ values from the respiratory gas analysis during the ramp test protocol on the treadmill (ramp protocol) and V̇O_2max_ values estimated by the smartwatch after run 1 (smartwatch run 1) and run 2 (smartwatch run 2) in all athletes. The bar represents the mean values (± SD) of all athletes; lines represent individual data of each athlete (*n* = 35)

Figure 2 shows the mean, standard deviations, and individually assessed V̇O_2max_ values from respiratory gas analysis during the ramp test, along with V̇O_2max_ estimates from the smartwatch after run 1 and run 2 for all athletes.

**Fig. 3:**
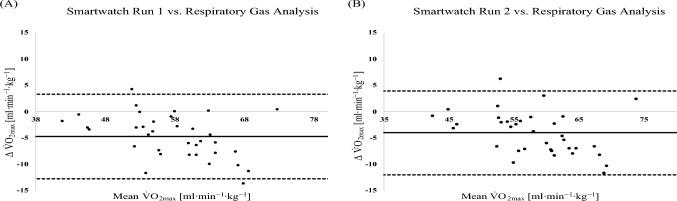
Bland–Altman analysis comparing the different V̇O_2max_ assessments in all athletes (*n* = 35). **A** smartwatch run 1 vs. respiratory gas analysis during ramp protocol on the treadmill. **B** Smartwatch run 2 vs. respiratory gas analysis during ramp protocol on the treadmill

Figure 3 presents the Bland–Altman analyses comparing V̇O₂_max_ values derived from the smartwatch and those obtained via respiratory gas analysis for the entire cohort. When comparing V̇O₂_max_ estimates from the first smartwatch run to the criterion measure obtained during the ramp test, the analysis revealed a mean bias of − 4.73 mL·kg⁻^1^·min⁻^1^, with 95% limits of agreement ranging from − 12.78 to 3.32 mL·kg⁻^1^·min⁻^1^. Similarly, the second smartwatch run demonstrated a mean bias of − 4.05 mL·kg⁻^1^·min⁻^1^, with 95% limits of agreement between − 12.02 and 3.93 mL·kg⁻^1^·min⁻^1^. These findings indicate a consistent underestimation of V̇O₂_max_ by the smartwatch relative to the gold standard, with a relatively wide range of individual differences.

#### .

### Moderately trained athletes with an V̇O_2max_ < 60 ml·min^−1^·kg.^−1^

**Fig. 4:**
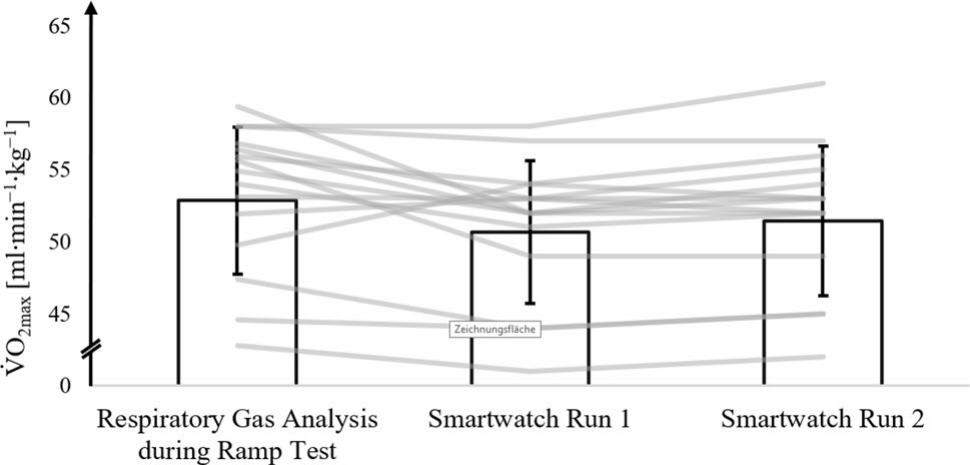
V̇O_2max_ values of moderately trained athletes with a V̇O_2max_ < 60 ml·min^−1^·kg^−1^ from the respiratory gas analysis during the ramp protocol on the treadmill (ramp protocol) and V̇O_2max_ values estimated by the smartwatch after run 1 (smartwatch run 1) and run 2 (smartwatch run 2). The bar represents the mean values (± SD) of moderately trained athletes with a V̇O_2max_ < 60 ml∙min^−1^·kg^−1^ (*n* = 18); lines represent individual data of each moderately trained athlete

Figure 4 illustrates the mean and standard deviations of V̇O_2max_ for moderately trained athletes with a V̇O_2max_ < 60 ml∙min⁻^1^∙kg⁻^1^, as assessed in the ramp test with respiratory gas analysis, and V̇O_2max_ estimates from the smartwatch after run 1 and run 2.

**Fig. 5:**
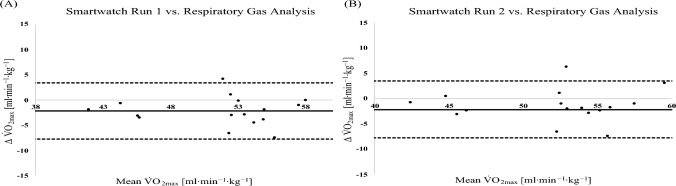
Bland–Altman analysis comparing the different V̇O_2max_ assessments in moderately trained athletes with a V̇O_2max_ < 60 ml∙min^−1^·kg^−1^ (*n* = 18). **A** Smartwatch run 1 vs. respiratory gas analysis during ramp test protocol. **B** Smartwatch run 2 vs. respiratory gas analysis during ramp test protocol

The Bland–Altman analysis comparing the different V̇O_2max_ assessments of moderately trained athletes with a V̇O_2max_ < 60 ml∙min^−1^·kg^−1^ is displayed in Fig. 5. The average difference and 95% limits of agreement revealed by the Bland–Altman analysis when comparing V̇O_2max_ values from smartwatch run 1 with the respiratory gas analysis and smartwatch run 2 with the respiratory gas analysis are − 2.17 ml·min^−1^·kg^−1^ (− 7.77 ml∙min^−1^·kg^−1^; 3.43 ml∙min^−1^·kg^−1^); -1.42 ml∙min^−1^·kg^−1^ (− 7.78 ml∙min^−1^·kg^−1^; 4.95 ml∙min^−1^·kg^−1^), respectively.

The Student’s t test comparing the two V̇O_2max_ assessments in moderately trained athletes revealed a significant difference between smartwatch- and respiratory gas analysis-derived V̇O₂_max_ values in run 1 (t = − 3.035, df = 15, *p* = 0.008, d = − 0.759), but not in run 2 (t = − 1.748, df = 15, *p* = 0.101, d = − 0.437).

### Highly trained athletes with a V̇O_2max_ > 60 ml·min^−1^·kg·^−1^

**Fig. 6:**
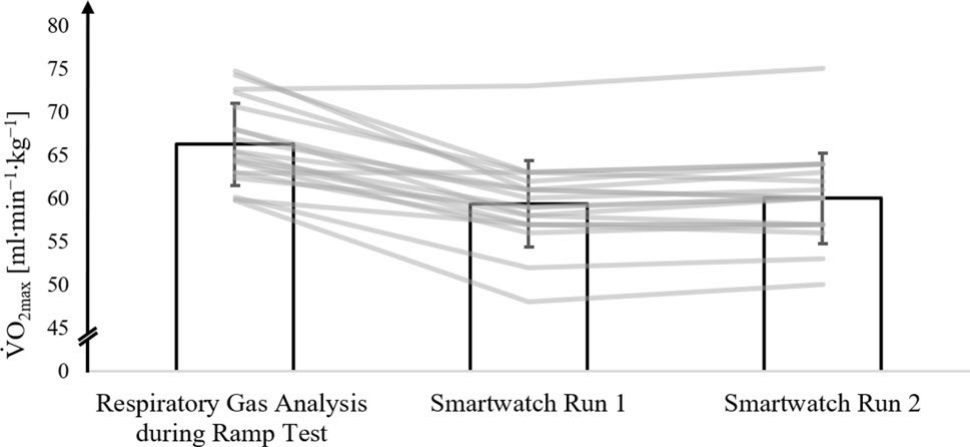
V̇O_2max_ values of each highly trained athlete with a V̇O_2max_ > 60 ml·min^−1^·kg^−1^ from the respiratory gas analysis during the ramp protocol on the treadmill (ramp protocol) and V̇O_2max_ values estimated by the smartwatch after run 1 (smartwatch run 1) and run 2 (smartwatch run 2). The bar represents the mean values (± SD) of highly trained athletes (*n* = 18); lines represent individual data of each highly trained athlete

Figure 6 displays the mean and standard deviations of V̇O_2max_ for highly trained athletes with V̇O_2max_ > 60 ml∙min⁻^1^·kg⁻^1^, as assessed in the ramp test with respiratory gas analysis, and V̇O_2max_ estimates from the smartwatch after run 1 and run 2.

**Fig. 7:**
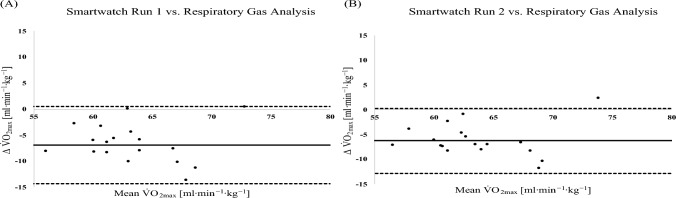
Bland–Altman analysis comparing the different V̇O_2max_ assessments in highly trained athletes with a V̇O_2max_ > 60 ml∙min^−1^·kg^−1^ (*n* = 18). **A** Smartwatch run 1 vs. respiratory gas analysis during ramp test protocol. **B** Smartwatch run 2 vs. respiratory gas analysis during ramp test protocol

The Bland–Altman analysis comparing the different V̇O_2max_ assessments of highly trained athletes with a V̇O_2max_ > 60 ml∙min^−1^·kg^−1^ are displayed in Fig. 7. The average difference and 95% limits of agreement revealed by the Bland–Altman analysis when comparing V̇O_2max_ values from smartwatch run 1 with the respiratory gas analysis and smartwatch run 2 with the respiratory gas analysis are − 6.88 ml·min^−1^∙kg^−1^ (− 14.31 ml∙min^−1^·kg^−1^; 0.53 ml∙min^−1^·kg^−1^); − 6.25 ml∙min^−1^·kg^−1^ (− 12.82 ml∙min^−1^·kg^−1^; 0.30 ml∙min^−1^·kg^−1^), respectively.

The Student’s t test comparing the two V̇O_2max_ assessments in highly trained athletes revealed a significant difference between smartwatch- and respiratory gas analysis-derived V̇O₂_max_ values in run 1 (t = − 7.936, df = 18, p < 0.001, d = − 1.821) and in run 2 (t = − 8.152, df = 18, *p* < 0.001, d = − 1.870).

Table 4 reports the intraclass correlation coefficients (ICC) and mean absolute percentage errors (MAPE) for the comparison between V̇O₂_max_ values obtained via respiratory gas analysis during the treadmill ramp protocol and those estimated by the smartwatch during the two 15-min outdoor runs. These metrics are presented for the entire sample as well as for the two stratified subgroups: moderately trained athletes with V̇O₂_max_ < 60 mL·kg⁻^1^·min⁻^1^ and highly trained athletes with V̇O₂_max_ > 60 mL·kg⁻^1^·min⁻^1^. This analysis enables an evaluation of both the relative agreement and the absolute estimation error between the two assessment methods across different levels of aerobic fitness.

**Table 4:** Intraclass correlation coefficient and the mean absolute percentage error for all athletes and the two subgroups (moderately trained athletes with a V̇O_2max_ < 60 ml∙min^−1^·kg^−1^ (*n* = 18) and highly trained athletes with V̇O_2max_ > 60 ml∙min^−1^·kg^−1^ (*n* = 17)) comparing two different methodologies assessing V̇O_2max_ (respiratory gas analysis during the ramp protocol on the treadmill and the two 15-min outdoor runs with the smartwatch)

	All athletes		Moderately trained athletes with V̇O_2max_ < 60 ml∙min^−1^∙kg^−1^		Highly trained athletes with V̇O_2max_ > 60 ml∙min^−1^∙kg^−1^	
	ICC [95% CI]; “Interpretation”	Mean absolute percentage error	ICC [95% CI]; “interpretation”	Mean absolute percentage error	ICC [95% CI]; “interpretation”	Mean absolute percentage error
Smartwatch run 1 vs. respiratory gas analysis	0.71 [0.03; 0.90] “moderate”	7.9	0.63 [0.09; 0.87] “moderate”	4.1	0.34 [− 0.11; 0.69] “poor”	10.4
Smartwatch run 2 vs. respiratory gas analysis	0.75 [0.17; 0.91] “good”	6.7	0.66 [0.21; 0.87] “moderate”	2.8	0.41 [-0.11; 0.75] “poor”	9.4

*ICC* intraclass correlation coefficient, *MAPE* mean absolute percentage error

## Discussion

Here, we aimed to validate the V̇O_2max_ estimation provided by a contemporary smartwatch in moderate vs. highly trained endurance athletes.

The main findings of the present study were as follows:

i) In the overall sample, smartwatch-derived V̇O₂_max_ values showed moderate-to-good agreement with criterion values obtained via respiratory gas analysis, with MAPE of 7.9% after the first run and 6.7% after the second run. However, the wide confidence intervals associated with the ICC suggest considerable variability in individual estimates. Additionally, the Student’s t test revealed significant differences in V̇O₂_max_ values between the two measurement methods in run 1 for both subgroups, and in run 2 for highly trained athletes, emphasizing the discrepancy between smartwatch-derived and respiratory gas analysis-derived V̇O₂_max_ values.
ii) Subgroup analyses revealed that moderately trained athletes demonstrated moderate agreement between smartwatch- and gas analysis-derived V̇O₂_max_ values, with a MAPE of 2.8% after run 2. In contrast, highly trained athletes exhibited poor agreement and a higher MAPE of 9.4% following two smartwatch-based assessments. For both subgroups, the wide ICC confidence intervals indicate that individual-level estimates may be prone to substantial error.
iii) Comparing V̇O₂_max_ estimates between the two smartwatch runs revealed improved validity across the entire sample, as well as within both subgroups. This improvement was evidenced by higher ICC values and lower MAPE in the second run, suggesting enhanced estimation accuracy with repeated use of the device. Additionally, the Student’s t test revealed a significant difference in V̇O₂_max_ values between the two measurement methods in run 1, but not in run 2 for moderately trained athletes.

Previous studies assessing the validity of the same smartwatch model used in the present study reported mean absolute percentage errors for smartwatch-derived V̇O₂_max_ estimates ranging from 1.06% to 5.58% (Düking et al. 2024) and from 5.6% to 5.7% (Düking et al. 2022) when compared to criterion measures obtained via respiratory gas analysis. Other studies evaluating V̇O₂_max_ estimates from devices produced by the same manufacturer have consistently reported MAPE values below 10%, indicating an overall acceptable level of agreement with laboratory-based reference methods (Passler et al. 2019; Carrier et al. 2020; Düking et al. 2022). The relatively low mean absolute percentage errors (MAPE < 10%) suggest that the smartwatch provides a reasonably accurate device for monitoring aerobic fitness trends over time in both recreational and well-trained populations, especially in contexts where laboratory-based testing is not feasible. However, users should be aware that smartwatch-derived V̇O₂_max_ estimates may differ from those obtained through standardized laboratory assessments, and this discrepancy should be considered when interpreting individual fitness levels.

A comparison of the smartwatch examined in the present study with devices from other manufacturers reveals notable variability in estimation accuracy. For instance, Passler et al. (Passler et al. 2019) reported a MAPE of 13.2% for a device produced by Polar Electro OY. Similarly, studies evaluating Fitbit devices observed MAPE values of approximately 10% (Klepin et al. 2019; Freeberg et al. 2019). In contrast, Caserman et al. (Caserman et al. 2024) found a higher MAPE of 15.79% for the Apple Watch Series 7. Collectively, these findings suggest that Garmin smartwatches may yield V̇O₂_max_ estimates with comparable or even lower error margins relative to other commercially available devices. Nevertheless, the inherent measurement inaccuracies of all wearable technologies must be acknowledged. When high precision is required—such as for clinical diagnostics, individualized training prescription, or scientific research—smartwatch-derived V̇O₂_max_ estimates should not be considered a substitute for criterion-based assessments using respiratory gas analysis during maximal ramp test protocols.

To enhance the validity of smartwatch-derived V̇O_2max_ estimations, our findings suggest that error rates decrease following a second outdoor run with the device. This observation aligns with the previous research conducted in soccer players (mean V̇O₂_max_: 56.6 ± 4.9 mL·kg⁻^1^·min⁻^1^), which reported a reduction in MAPE after a second running session using the same smartwatch model (Düking et al. 2024). Conversely, another study employing the same device did not observe a reduction in error rates of V̇O_2max_ following repeated assessments (Düking et al. 2022). Although the cause of this discrepancy remains unclear, one plausible explanation may lie in algorithmic changes implemented through undisclosed firmware or software updates by the manufacturer, which may have influenced the V̇O₂_max_ estimation process across different study periods. Additionally, we cannot entirely rule out that the improved accuracy of smartwatch-derived V̇O₂_max_ estimation following the second run may, in part, be influenced by subtle adjustments in pacing or effort by the participants. However, the comparable heart rate and distance values recorded across both runs, in conjunction with the good intraclass correlation coefficients (ICCs; see Table 3), indicate that participants performed both runs at a similar intensity level.

We identified large confidence intervals for the error rates when assessing V̇O_2max_ with the smartwatch (ICC = 0.75 [95% CI: 0.17; 0.91] after 2 runs) and as visible in the respective Bland–Altman plots. Large 95% confidence intervals have been reported in the previous studies on the respective smartwatch (Düking et al. 2024, 2022). These results suggest that individual V̇O_2max_ estimations derived from the smartwatch may be associated with considerable error. Therefore, practitioners and researchers are advised to interpret such measurements with caution and, where possible, verify them against criterion methods, particularly when precise assessments are required.

### Highly trained athletes vs. moderately trained athletes

Given prior evidence suggesting that the accuracy of smartwatch-derived V̇O₂_max_ estimates decreases in highly trained individuals with V̇O_2max_ values exceeding 60 mL·kg⁻^1^·min⁻^1^ (Düking et al. 2024), we stratified our sample accordingly and conducted a subgroup analysis. Participants were categorized into highly trained athletes (V̇O_2max_ > 60 mL·kg⁻^1^·min⁻^1^) and moderately trained athletes (V̇O₂_max_ ≤ 60 mL·kg⁻^1^·min⁻^1^). As hypothesized, the highly trained subgroup, characterized by a true V̇O₂_max_ of 67.0 ± 4.3 mL·kg⁻^1^·min⁻^1^, demonstrated a greater estimation error (MAPE = 9.4%) and lower agreement with the criterion measure (ICC = 0.41 [95% CI: − 0.11 to 0.75]) compared to the moderately trained subgroup, whose true V̇O₂_max_ was 53.6 ± 5.0 mL·kg⁻^1^·min⁻^1^ (MAPE = 2.8%; ICC = 0.66 [95% CI: 0.21 to 0.87]). Notably, these differences in estimation accuracy persisted even after two standardized outdoor runs with the smartwatch.

To the best of our knowledge, only one previously published study assessed the validity of smartwatch-derived V̇O_2max_ estimations vs. laboratory-based V̇O_2max_ diagnostics in trained individuals with a laboratory-based V̇O_2max_ of approx. 60 ml·min^−1^·kg^−1^ (Carrier et al. 2023). The authors assessed another model of the herein tested manufactured and concluded that the smartwatch-derived V̇O_2max_ estimation had a mean absolute percentage error of 6.85% versus the laboratory-based V̇O_2max_ (Carrier et al. 2023). The mean absolute percentage error observed in our study for the highly trained subgroup was considerably higher—10.4% after run 1 and 9.4% after run 2. This discrepancy may be attributable to the higher actual V̇O₂_max_ observed in our participants (67.0 ± 4.3 mL·kg⁻^1^·min⁻^1^) compared to the mean V̇O₂_max_ of 57.88 mL·kg⁻^1^·min⁻^1^ reported by Carrier et al. (2023), which was calculated as a 30-s average. These findings further support the notion that smartwatch-based estimation algorithms may be less accurate in individuals with exceptionally high aerobic capacities.

We observed a poor ICC along with large 95%CI in our subgroup analysis of highly trained athletes (ICC = 0.41 [95%CI: -0.11; 0.75]) for the agreement between smartwatch-derived and respiratory gas analysis-derived V̇O_2max_. This suggests that V̇O_2max_ estimations from smartwatches in highly trained individuals, with true values around 60–70 ml·min^−1^·kg^−1^ or higher, should be interpreted with great caution.

The MAPE of 2.8% observed in our study (after two outdoor runs) for moderately trained athletes with a true V̇O₂_max_ of 53.6 ± 5.1 mL·kg⁻^1^·min⁻^1^ is consistent with the previous research on smartwatch-derived V̇O₂_max_ estimations. Notably, a prior study conducted by our group reported an even lower MAPE of 1.06% following two outdoor runs in individuals with a similar aerobic capacity (V̇O₂_max_: 56.6 ± 4.9 mL·kg⁻^1^·min⁻^1^), as assessed via respiratory gas analysis. These findings suggest that smartwatch-based V̇O₂_max_ estimations may yield acceptable levels of accuracy in individuals with V̇O₂_max_ < 60 mL·kg⁻^1^·min⁻^1^, particularly when multiple measurement sessions are used. (Düking et al. 2024).

### Strengths and limitations

A notable strength of the present study is the inclusion of highly trained endurance athletes (mean V̇O₂_max_: 60.1 ± 8.2 mL·kg⁻^1^·min⁻^1^; range: 59.8–74.7 mL·kg⁻^1^·min⁻^1^) and the stratification of data based on V̇O₂_max_ values above or below 60 mL·kg⁻^1^·min⁻^1^. This subgroup analysis enabled more targeted insights into the validity of smartwatch-derived V̇O₂_max_ estimates across different levels of aerobic fitness, an area that remains underexplored in the literature. However, the inclusion of multiple smartwatch models from the same manufacturer, as well as an analysis of inter-device reliability (i.e., different units of the same smartwatch model), could have allowed for a more comprehensive assessment of the variability and reliability of smartwatch-derived V̇O₂_max_ estimations in athletes. A further limitation of this study is the gender imbalance within the sample. Despite efforts to achieve equal representation, fewer female athletes were recruited, reflecting the generally lower participation of women in endurance sports and associated training environments. Although sex-related effects were not the focus of this study and are not expected to have substantially influenced the results, their potential impact cannot be entirely excluded.

Given the observed improvements in validity-related metrics—such as reduced mean absolute percentage error and higher intraclass correlation coefficients—after two 15-min outdoor runs with the smartwatch (with GPS and heart rate sensors activated), it is plausible that further enhancements in estimation accuracy may be achievable with additional or prolonged measurement sessions. Future research should explore whether extended or repeated submaximal runs can improve the precision of smartwatch-derived V̇O₂_max_ estimates, particularly in highly trained populations.

It is important to acknowledge that the present findings are specific to the smartwatch model (Forerunner 245), the software version used, and the cardiorespiratory fitness range of the study cohort. V̇O₂_max_ estimations may differ across smartwatch models, firmware updates, or user characteristics. Since manufacturers of consumer-grade devices are not required to disclose algorithmic changes in detail, future software or device versions may yield different results. Ongoing validation research is therefore warranted and would benefit substantially from increased transparency by manufacturers regarding algorithm updates and data processing procedures.

## Conclusions

Our findings demonstrate that when two 15-min outdoor runs are performed at an intensity exceeding 70% of HRₘₐₓ using the tested smartwatch, a mean absolute percentage error (MAPE) of 6.7% in V̇O₂_max_ estimation can be expected. However, subgroup analyses revealed a clear dependency on fitness level: in highly trained athletes (mean V̇O₂_max_: 67.0 ± 4.3 mL·kg⁻^1^·min⁻^1^), the MAPE increased to 9.4%, whereas in moderately trained individuals (mean V̇O₂_max_: 53.6 ± 5.1 mL·kg⁻^1^·min⁻^1^), the MAPE was substantially lower at 2.8%.

Given these error margins, and particularly the wide confidence intervals observed in our statistics, smartwatch-derived V̇O₂_max_ estimates should be interpreted with caution. While such devices may provide a convenient and reasonably accurate estimate for recreational and moderately trained individuals, they are not a substitute for laboratory-based assessments. When precise measurement is required—for instance, in elite athlete monitoring or scientific research— V̇O₂_max_ should be assessed using gold-standard methods involving respiratory gas analysis during maximal exertion protocols.

## Data Availability

The datasets generated and/or analyzed during the current study are available from the corresponding author on reasonable request.
